# Supplementary material for: A whole-animal phenotypic drug screen identifies suppressors of atherogenic lipoproteins
Source: eLife. 2026 Aug 27;14:RP105314. doi: 10.7554/eLife.105314 (PMC13521566; doi:10.7554/eLife.105314)
Supplement: Supplementary file 2 [file elife-105314-code1.zip › Output_data/ranked_lomi_SSMD.pdf]

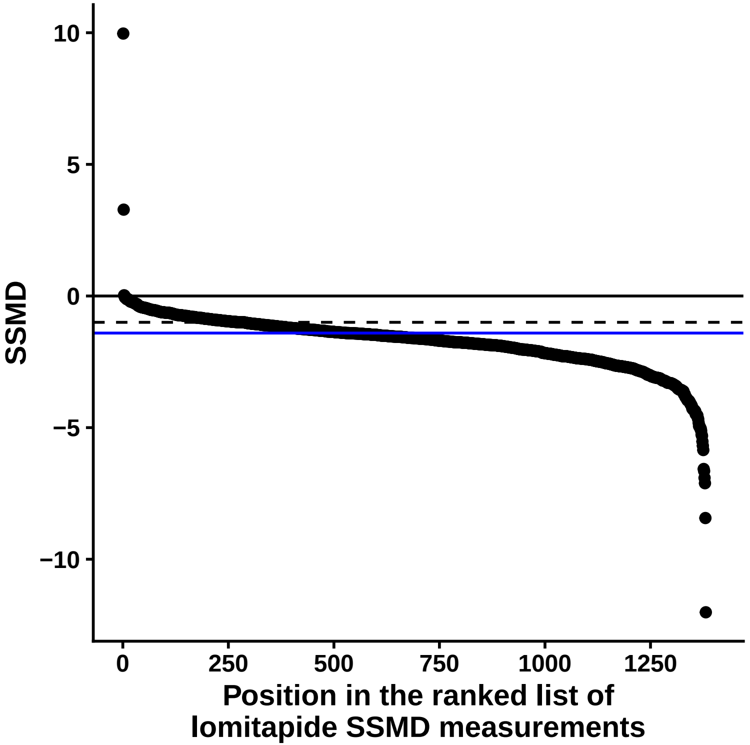

10
5
0
−5
−10
SSMD
0
250
500
750
1000
1250
Position in the ranked list of
lomitapide SSMD measurements
